# The emerging role of astrocytes in spatial cognition, action and Alzheimer’s disease

**DOI:** 10.3389/fncel.2026.1811370

**Published:** 2026-07-27

**Authors:** Marion Gaborit, Anahita Arabi, Letizia Mariotti

**Affiliations:** Institute of Neuroscience, National Research Council (CNR), Padova, Italy

**Keywords:** Alzheimer’s disease, astrocytes, goal-oriented behaviour, neuro-glia interaction, spatial memory

## Abstract

Astrocytes sense synaptic activity, neuromodulation, and metabolic signals, and respond by modulating neurotransmission, excitability, and plasticity in neural networks. Increasing evidence demonstrates that these processes contribute to information processing and encoding across brain areas, thereby influencing perception, memory, cognition, and goal-directed behaviours. Notably, impairments in these functions represent some of the earliest hallmarks of ageing and neurodegenerative disorders such as Alzheimer’s disease. Here we review molecular, cellular, and computational insights into astrocyte-neuron interactions, framing their importance at the system and behavioural level; then, we discuss the role of these interactions in spatial cognition, goal-directed behaviour, and their impairment in Alzheimer’s disease, and review the mouse models used to investigate spatial deficits and associated astrocyte activity. Finally, we highlight major open questions in the field, outline future research directions, and discuss emerging strategies to address astrocyte role in cognitive function in health and disease.

## Introduction

A leading theory posits that astrocytes are active drivers of information processing in neural networks (Perea and Araque, 2005; Benoit et al., 2025), able to sense neurotransmission and metabolic demand (Suzuki et al., 2011) and respond by modulating neuronal activity and plasticity (Fellin et al., 2004; Perea and Araque, 2007; Panatier et al., 2011). This theory is corroborated by decades of work suggesting that astrocytes could encode different patterns of neurotransmission and neuromodulation in distinct spatio-temporal dynamics intracellular Ca^2+^ signalling (Perea and Araque, 2005; Semyanov et al., 2020). The ensuing intracellular cascades can trigger the release of gliotransmitters (Pascual et al., 2005; Henneberger et al., 2010; de Ceglia et al., 2023) and regulate neurotransmitter uptake (Oliet et al., 2001; Murphy-Royal et al., 2015), which in turn modulate response gain (Perea et al., 2014), network synchronisation (Fellin et al., 2004; Poskanzer and Yuste, 2016), synaptic efficacy (Jourdain et al., 2007; Panatier et al., 2011), and plasticity (Henneberger et al., 2010; Volterra et al., 2014). Moreover, by coupling neuronal activity to metabolic (Suzuki et al., 2011) and vascular responses (Takano et al., 2006; Mishra et al., 2016), astrocyte can further influence local and global function across brain networks (Barnett et al., 2023; Reitman et al., 2023). Thus, while further work is needed to establish their causal role in information processing, an emerging hypothesis is that astrocytes could expand the repertoire of neural computations (De Pitta et al., 2012).

In this framework, not only neurons, but also astrocytes could influence cognition (Curreli et al., 2022), memory (Navarrete et al., 2012) and goal-directed behaviours (Mederos et al., 2021; Nagai, Yu et al., 2021b; Sánchez Romero and Navarrete, 2026). An increasing number of studies reveal specialised functions and heterogeneity of astrocytes across brain areas. In the striatum, astrocytes actively participate in action selection and reward-seeking (Kang et al., 2020); in the prefrontal cortex, astrocyte inhibition alters cortical oscillations and impairs goal-directed behaviour (Mederos et al., 2021); in the hippocampus, astrocytes support neuronal encoding of position and salient spatial locations (Curreli et al., 2022; Doron et al., 2022). Together, these findings suggest that different astrocytes serve specialised functions and are integral components of the neuronal circuits driving spatial coding and goal-directed actions.

Notably, deterioration of memory and goal-directed behaviours represents one of the earliest hallmarks of ageing and neurodegenerative disorders such as Alzheimer’s disease (AD; Coughlan et al., 2018). An emerging perspective is that astrocyte dysfunction could prime or contribute to circuit dysregulation leading to deficits in spatial navigation, action selection, and cognitive flexibility (Santello et al., 2019). Understanding how astrocytic alterations translate into network dysfunction and behavioural impairment may therefore open new avenues for astrocyte-targeted therapeutic interventions aimed at restoring cognitive function.

In this mini-review, we integrate molecular, cellular, and computational insights into astrocyte–neuron interactions within a behavioural and systems-level framework to examine how these interactions support navigation and goal-directed behaviour. We then review evidence from mouse models of AD (Table 1) and discuss emerging strategies to better characterise and modulate astroglia contributions to circuit function. Finally, we propose an integrated conceptual framework in which spatial cognition emerges from astrocyte-neuronal units, positioning astrocytes as active participants in memory, decision-making, and adaptive behaviour. We also highlight open questions and future experimental avenues to address them.

**Table 1:** Relevant AD mouse models showing astrocyte impairments, memory loss and spatial deficit.

Mouse model	Pathology onset	Main impairments	Brain area	Behavioural tests	References
APPPS1	Aβ plaques~4mo. Cognitive deficits~6mo.	Late reversal learning and memory deficits ↓Astrocytic Ca^2+^ transients and astrogliosis Aβ-induced astrocytes reactivity Excessive astrocytic GABA production Synaptic dysfunctions Neurodegeneration	Cortex HPC	MWM T-maze Four-arm RAM CFC Open field	Jo et al. (2014); Orr et al. (2015); Orr et al. (2018); Karunakaran (2020); Lee et al. (2023); Hamilton et al. (2023)
APPPS2	Aβ plaques~6mo. Cognitive deficits ~9-10mo.	Spatial memory deficits Reactive astrocytes and gliosis Impaired astrocyte Ca^2+^ dynamics Impaired astrocyte-neuron interactions Neurodegeneration	Cortex (SSCx) HPC (CA1)	MWM Y-maze ORT Tactile ORT AAT	Richards et al. (2003); Fontana et al. (2017); Leparulo et al. (2019); Lia et al. (2023)
3xTg-AD	Aβ plaques~6mo. Tau tangles~12mo. Cognitive deficits ~7-12mo.	Spatial memory deficits Metabolic stress (↑astrocytic Kir6.2-IR) Lower NMDAR occupancy (↓L/D-ser) Astrogliosis and Ca^2+^ signalling deficits Impaired NT clearance Early synaptic impairments Neurodegeneration	Cortex (SSCx) HPC (CA1)	MWM EPM NOR OC	Oddo et al. (2003); Griffith et al. (2016); Belfiore et al. (2019); Le Douce et al. (2020); Duggan et al. (2024)
5xFAD	Aβ plaques~3-5mo. Limited Tau tangles Cognitive deficits~4-6mo.	Object location memory deficits HPC-related short-term memory deficits Long term retention memory deficits Oxidative stress (↓Nrf2) Mitochondrial damage Astrocytes reactivity and astrogliosis Impaired neuronal Ca^2^ signalling Neurodegeneration	Cortex HPC Limbic System	MWM EPM Open field OLT PAT	Oakley et al. (2006); Nakano-Kobayashi et al. (2023); Huang et al. (2020)
Tg2576	Aβ plaques~12mo. Cognitive deficits ~12mo.	Late spatial learning and memory deficits Working memory impairment Apoptosis and neuroinflammation Early astrocytes reactivity (↑K_V_3.4) ↑Aβ trimer levels Neurodegeneration	Cortex HPC Cerebellum	MWM T-maze Y-maze RAWM Probe test PAT	Webster et al. (2014); Boscia et al. (2017); Ham et al. (2020)
APP NL-G-F Knock-in	Aβ plaques~6mo. Cognitive deficits~6mo.	Severe memory impairments Reactive astrocytes Neuroinflammation High levels of astrocytosis Synaptic deficits (↓synaptophysin and PSD95)	Cortex HPC	Y-maze	Saito et al. (2014)
AppNL-G-F/MAPT double knock-in	Aβ plaques~6mo. High levels of phosphorylated Tau~12mo. Cognitive deficits~6mo.	Hippocampal atrophy Memory impairments Astrogliosis Ca^2+^ homeostasis dysregulations NMDAR-induced cytotoxicity Mitochondrial dysfunctions Plasticity deficits Neurodegeneration	Cortex HPC	Y-maze	Saito et al., 2014; Hashimoto et al. (2019)
AD-BXD (5xFAD × BXD)	Aβ plaques~4-6mo. Cognitive deficits~6-14mo.	Short-term and long-term memory deficits Working memory impairments Astrocyte-specific lipid and cholesterol pathways dysregulations Reactive astrocytes Neuroinflammation Impaired neuron-astrocyte metabolic support Synaptic dysfunctions Neurodegeneration	Cortex HPC	Y-maze CFC	Neuner et al. (2019); Heuer et al. (2020); Gurdon et al. (2024)

The table provides a general overview of transgenic mouse models of AD presenting amyloid-β plaque deposition and associated with astrogliosis. To our knowledge, these models have been evaluated for astrocyte-related impairments, as well as for spatial memory and navigation deficits. For each model, the table reports: pathological onset; predominant astrocyte-related cognitive and memory impairments; the most affected brain regions; and key behavioural paradigms used to detect these deficits. AAT, active avoidance test; AD, Alzheimer’s disease; APP, amyloid precursor protein; CFC, contextual fear conditioning; EPM, elevated plus maze; FAD, familial Alzheimer’s disease; Four-arm RAM, four-arm radial arm maze; HPC, hippocampus; MWM, Morris water maze; NOR, Novel object recognition; OC, operant conditioning; OLT, object location test; ORT, object recognition test; PAT, passive avoidance test; PS1/2, Presenilin-1/2; RAWM, radial arm water maze; SSCx, somatosensory cortex.

### Astrocytes as specialised integrators and modulators of neuronal activity and plasticity

Astrocytes are characterised by a ramified, complex morphology, with intricate fine processes closely associated with neuronal synapses (Ventura and Harris, 1999; Bushong et al., 2002). This distinctive anatomical organisation was termed *tripartite synapse* (Araque et al., 1999; Perea et al., 2009), positioning astrocytes as integral components of synaptic circuits able to directly monitor and modulate synaptic activity (Perea and Araque, 2005; Santello et al., 2019; Kofuji and Araque, 2021; Barnett et al., 2023; Benoit et al., 2025). In this framework, astrocytes tile the entire neuropil by occupying largely non-overlapping territorial domains, approximately 100–120 μm in diameter, within which each astrocyte enwraps multiple synapses (Halassa et al., 2007). In the neocortex, it is estimated that the fraction of cortical synapses in contact with astrocytic processes is around ~30% (Spacek, 1985; Bernardinelli et al., 2014), while in the hippocampus, dendritic spines in direct apposition to astrocytes rise to ~60–90% of the total (Witcher et al., 2007; Reichenbach et al., 2010; Bernardinelli et al., 2014).

Within their individual domains, astrocytes are therefore ideally positioned to detect and possibly integrate multiple, potentially diverse synaptic inputs (Perea and Araque, 2005; Benoit et al., 2025). Electron microscopy studies have revealed that astrocytic fine processes express a complement of proteins involved in synaptic transmission (Chai et al., 2017; Aboufares El Alaoui et al., 2021), enabling astrocytes to sense different neurotransmitters released from both pre- and post-synaptic elements (Fellin et al., 2004; Panatier et al., 2011; Mariotti et al., 2018). In response, astrocytes exhibit dynamic intracellular Ca^2+^ signals, which can trigger the release of gliotransmitters and activate signalling pathways that modulate synaptic efficacy and plasticity (Mariotti et al., 2016; Cahill et al., 2024).

The fluctuation of intracellular Ca^2+^ concentration is at the basis of astrocytes excitability (Perea and Araque, 2005; Di Castro et al., 2011; Bindocci et al., 2017; Semyanov et al., 2020). These dynamics are primarily driven by the activation of G protein–coupled receptors (GPCRs) (Kofuji and Araque, 2021) and the subsequent Ca^2+^ release from intracellular stores via inositol 1,4,5-trisphosphate (IP₃)-dependent signalling pathways (Sherwood et al., 2021; Lines et al., 2024). Ca^2+^ elevations can arise spontaneously, as intrinsic or stochastic oscillations (Nett et al., 2002; Wang et al., 2006; Losi et al., 2025), or can be triggered in response to neuronal activity and synaptic transmission (Stobart et al., 2018; Lines et al., 2024).

The spatio-temporal pattern of these intracellular Ca^2+^ signals depends on the complexity of their morphology and active propagation mechanism, resulting in spatial dynamics respecting different temporal scales and thresholds (Rupprecht et al., 2024). Spatially, Ca^2+^ signals may remain local to small subcellular compartments, termed *microdomains* (Di Castro et al., 2011; Shigetomi et al., 2013; Bindocci et al., 2017), invade whole branches, or recruit a whole-cell Ca^2+^ event, commonly termed as *Ca^2+^ surge* (Lines et al., 2024). Oversimplifying, this propagation appears to follow a spatial integration rule: when Ca^2+^ activity engages approximately 25% of an astrocyte’s arborization within a restricted temporal window, the signal propagates toward the soma and then spreads across the entire cell, triggering a Ca^2+^ surge (Lines et al., 2024; Rupprecht et al., 2024). Below this spatial threshold, Ca^2+^ activity remains instead local (Araque et al., 2014; Srinivasan et al., 2015; Bindocci et al., 2017; Lines et al., 2024).

Thus, rather than passive responders, astrocytes may operate as spatial and temporal integrators of neuronal signals (Perea and Araque, 2005; Araque et al., 2014; Benoit et al., 2025). The dynamics of microdomains and surges correspond to the summation of local and compartmentalised signal events into global responses. From a temporal perspective, small, fast, and highly localised Ca^2+^ transients within microdomains reflect the activation of single or few synapses, while global surges, triggered by synchronous activation of many synapses, encode slower timescale variations of network activity (Di Castro et al., 2011; Panatier et al., 2011; Kofuji and Araque, 2021). This framework suggests two modes of communication: microdomain activity could represent the fundamental units of astrocytic signalling, local and fast; global surges would instead correspond to global, integrative outputs triggered by the coordinated activation of multiple microdomains (Araque et al., 2014; Goenaga et al., 2023; Lines et al., 2024; Rupprecht et al., 2024). The spatial and temporal rules of integration of these signals remain largely unknown, highlighting the need to fill fundamental gaps in our understanding of the astrocyte signalling code, and how it governs synaptic transmission, plasticity and memory.

Astrocyte activation can trigger local or global gliotransmission, which in turn can bidirectionally modulate neuronal activity, learning and behaviour in a region-specific manner (Petrelli and Bezzi, 2016; Sánchez Romero and Navarrete, 2026). Upon Ca^2+^ elevation, astrocytes can release a variety of gliotransmitters, such as glutamate (Angulo et al., 2004), adenosine (Ado)/ATP (Pascual et al., 2005), and D-serine (Henneberger et al., 2010), which exert a wide range of functional effects on neurons (Mariotti et al., 2016). For example, the activation of the Gq cascade in hippocampal astrocytes promotes synaptic plasticity through a D-serine-dependent modulation of NMDA receptors (NMDARs) at CA3–CA1 synapses, selectively boosting activity in memory-encoding neuronal ensembles, thus enhancing spatial and contextual memory (Yang et al., 2003; Henneberger et al., 2010; Papouin et al., 2012, 2017; Adamsky et al., 2018; Covelo and Araque, 2018; Mederos et al., 2019). Similar astrocyte activation in the central amygdala instead suppresses neuronal firing via ATP/Ado release and reduces fear responses (Martin-Fernandez et al., 2017).

Further demonstration of astrocyte involvement in cognition comes from loss-of-function approaches. Deletion of astrocytic cannabinoid receptor type 1 (CB1Rs) or nuclear factor IA (NFIA) impairs astrocyte Ca^2+^ signalling and D-serine availability, leading to decreased hippocampal synaptic plasticity and recognition memory (Robin et al., 2018; Huang et al., 2020). Likewise, disrupting astrocyte gliotransmission using dnSNARE or tetanus toxin models alters the hippocampus-prefrontal cortex theta synchrony, disrupting multiple memory aspects; effects that can be rescued by D-serine application (Pascual et al., 2005; Lee et al., 2014; Sardinha et al., 2017). Finally, perturbation of the astrocytic Gs-GPCR signalling is strongly implicated in memory deficits associated with ageing and AD, and modulating this pathway can affect cognitive performance (Orr et al., 2015; Adamsky et al., 2018; Nagai, Bellafard et al., 2021a; Rupprecht et al., 2024).

These results support the idea that astrocytes are indeed a genetically heterogeneous class of cells whose molecular identity (Batiuk et al., 2020) and functional specialisation determine selective recruitment in different brain regions (Endo et al., 2022; Dewa et al., 2025), leading to circuit-specific outcomes (Adamsky et al., 2018; Kang et al., 2020; Sun et al., 2024). This heterogeneity appears not only across areas, but also within the same brain region, where different ensembles of astrocytes can be differentially engaged during learning. The emerging concept of *astroengrams* (Sánchez Romero and Navarrete, 2026), provide a unifying model to understand how astrocytes reciprocally communicate with neurons to shape and stabilise memory traces: rather than acting independently, astrocytes and neurons form integrated, coordinated networks whose joint activity shapes the engrams underlying memory-related behaviour. We suggest that the same astrocyte-dependent mechanisms that govern synaptic plasticity and engram formation are likely to influence the neuronal computations underlying goal-directed actions, particularly those requiring the integration of past experiences with action-outcome contingencies and behavioural flexibility.

### Astrocyte in spatial coding and goal-directed behaviours

Goal-directed reward-based behaviour provides a powerful framework to investigate the contribution of astrocytes to cognitive functions. The ability to learn and update spatial representations of the environment is fundamental and evolutionarily conserved across species. Animals must locate sources of food and shelter while avoiding potential threats. Such goal-directed actions depend on the coordinated activity of multiple brain regions that encode the spatial relationships among the self, objects, and goals within the environment. Through these processes, the brain generates, monitors, and updates motor plans and cognitive maps that enable adaptive behaviour in dynamic environments (O’Keefe, 1976; Moser et al., 2017; Olson et al., 2020).

Recent *in vivo* studies revealed that astrocytes directly contribute to spatial information processing in the hippocampus, encoding position and salient spatial locations. Two-photon Ca^2+^ imaging in mice navigating a reward-based virtual environment showed that CA1 astrocytes exhibit increasing activity as animals approach a reward in a familiar spatial context, identifying astrocyte signalling as a contributor to context-dependent spatial encoding (Doron et al., 2022). Astrocyte Ca^2+^ signals also encode spatial information in a topographically organised and compartment-specific manner, providing spatial information that is synergistic with, yet distinct from, that encoded by neurons (Curreli et al., 2022). Mechanistically, astrocyte contributions to spatial representations are likely to be closely linked to synaptic plasticity. Astrocyte activity in CA1 is sufficient to drive *de novo* synaptic potentiation and enhance spatial memory, indicating a direct role for astrocytes in plasticity (Adamsky et al., 2018). Consistent with this view, astrocytic CB1R signalling gates NMDAR-dependent plasticity by controlling D-serine availability at CA1 synapses (Robin et al., 2018; Bohmbach et al., 2022). These processes are further shaped by neuromodulatory inputs: noradrenergic projections from the locus coeruleus promote the propagation of astrocytic Ca^2+^ signals during behaviourally relevant events (Rupprecht et al., 2024). Beyond local synapses, astrocytes influence memory processing also at a circuit level. Hippocampal astrocytes regulate spatial memory by modulating projections to the anterior cingulate cortex engram neurons (Refaeli et al., 2024), while medial prefrontal cortex astrocytes support memory consolidation through Ado signalling (Iwai et al., 2021).

Coordinated activity across distinct astrocyte ensembles supports memory traces across modalities and areas. Three independent studies demonstrated that astrocyte ensembles from the hippocampus, the amygdala and the nucleus accumbens are both sufficient and necessary for memory recall (Williamson et al., 2024; Dewa et al., 2025; Serra et al., 2025). The reactivation of fear learning-associated astrocytes in the hippocampus is sufficient to elicit memory recall, establishing a causal role of astrocytes in memory expression (Williamson et al., 2024). In parallel, fear conditioning recruits astrocyte ensembles in the basolateral amygdala; silencing these recall-activated astrocytic ensembles disrupts memory stability during subsequent recall (Dewa et al., 2025). Similarly, astrocytic ensembles in the nucleus accumbens are specialised in the formation of cue–reward associations. Bidirectional manipulation of these ensembles causally altered cue–reward associations, directly implicating astrocytes in motivational memory processes (Serra et al., 2025).

**Figure 1:**
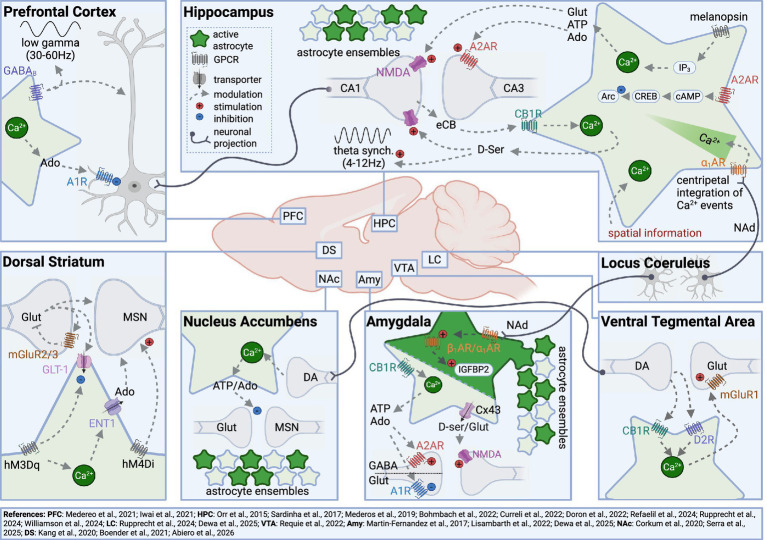
Astrocyte modulation of spatial coding and goal-directed behaviour across key brain regions and mechanisms. In the prefrontal cortex (PFC), astrocyte Ca^2+^ signalling triggers adenosine (Ado) release, activating neuronal adenosine A1 receptors (A1Rs) and inhibiting neuronal activity. Astrocytic GABA_B_ receptors also modulate neuronal firing and low-gamma oscillations. PFC engram neurons are influenced by hippocampal CA1 inputs under astrocytic modulation. In the hippocampus (HPC), astrocytes regulate CA3–CA1 transmission through IP_3_-dependent Ca^2+^ elevations that trigger glutamate, ATP, and adenosine release, engaging NMDA and adenosine A2A receptors (A2ARs) to promote synaptic plasticity. Endocannabinoid (eCB) signalling activates astrocytic cannabinoid type 1 receptors (CB1Rs) and Ca^2+^-dependent D-serine (D-ser) release, facilitating NMDA-dependent transmission, and HPC-PFC theta synchrony. Astrocytic A2AR-cAMP-CREB signalling downregulates Arc, a key mediator of memory consolidation. Astrocytes in CA1 also encode spatial information and form learning-associated astrocyte ensembles. CA1 astrocyte Ca^2+^ activity is shaped by noradrenergic (NAd) locus coeruleus (LC) input, integrated as Ca^2+^ signals propagating from processes to the soma. In the ventral tegmental area (VTA), dopaminergic (DA) neuron activity recruits astrocytic CB1Rs and dopamine D2 receptors (D2Rs), elevating Ca^2+^ signalling and metabotropic glutamate receptor 1 (mGluR1)-dependent plasticity. In the amygdala (Amy), noradrenergic inputs from the locus coeruleus support memory consolidation by activating astrocyte ensembles and upregulating β1- and α1-adrenergic receptors (β1ARs, α1ARs), increasing insulin-like growth factor-binding protein 2 (IGFBP2) expression. Astrocytes also modulate plasticity via connexin 43 (Cx43) hemichannel-mediated glutamate and D-serine (D-ser) release onto NMDA receptors and via CB1R-driven Ca^2+^ signalling that promotes ATP and adenosine release, differentially regulating glutamatergic and GABAergic transmission through adenosine A2ARs and A1Rs. In the nucleus accumbens (NAc), DA input elevates astrocytic Ca^2+^, promoting ATP/Ado release and synaptic depression, while astrocyte ensembles encode cue–reward associations. In the dorsal striatum (DS), astrocytes modulate goal-directed action. Gq-coupled hM3Dq receptors induce equilibrative nucleoside transporter 1 (ENT1)-mediated Ado release affecting direct (D1) and indirect (D2) medium spiny neurons (MSN) and modulate glutamate transporter 1 (GLT-1) and mGluR2/3 feedback to support behavioural flexibility. Here, Gi-coupled hM4Di receptor activation increases MSN activity. Relevant articles describing each brain region are reported in the reference box. Part of the schemes presented in Figure 1 were created with BioRender.com.

Beyond spatial memory representations, astrocytes play a central role in goal-directed behaviours. In the striatum, astrocytes actively participate in action selection and reward-seeking. In the dorsomedial striatum, Gq-mediated activation of astrocytic signalling promotes a shift from habitual to goal-directed reward-seeking behaviour (Hong et al., 2020; Kang et al., 2020; Boender et al., 2021; Serra et al., 2025; Abiero et al., 2026). This effect is associated with changes in the electrophysiological properties of medium spiny neurons (MSNs) and regulated by Ado signalling via the equilibrative nucleoside transporter 1 (ENT1) (Kang et al., 2020). Similarly, upregulation of astrocytic ENT1 in the dorso-medial striatum (DMS) promotes flexibility in ethanol-seeking behaviours (Hong et al., 2020). Conversely, Gi-mediated activation of astrocyte signalling in DMS impairs goal-directed action control and increases MSN activity, highlighting a bidirectional influence of astrocytes on neuronal circuits required for action-outcome learning (Abiero et al., 2026). In the dorso-lateral striatum, reduced behavioural flexibility is associated with increased astrocytic glutamate transporter 1 (GLT-1) expression, leading to enhanced glutamate clearance and altered glutamatergic neurotransmission (Boender et al., 2021). Astrocyte ensembles modulate cue-reward associations also in the nucleus accumbens (Corkrum et al., 2020; Serra et al., 2025) and in the medial prefrontal cortex, where astrocyte GABA_B_ receptor modulation perturbs neuronal activity and low gamma oscillation, ultimately affecting goal-directed behaviours (Mederos et al., 2021). More broadly, goal-directed behaviours rely on the integration of motivational and salience signals, classically associated with neuronal circuits in the ventral tegmental area and the amygdala (Balleine and O’Doherty, 2010). In these regions, astrocytes modulate neuronal activity and behaviour (Martin-Fernandez et al., 2017; Corkrum et al., 2020; Linsambarth et al., 2022; Requie et al., 2022) underlining their role in regulating motivational and salience-processing circuits relevant for goal-directed actions (for a summary of key regions and mechanisms of astrocyte signalling involved in spatial coding, navigation and goal-directed behaviour, see Figure 1).

Together, these findings indicate that astrocytes are active, context-dependent regulators of synaptic and circuit mechanisms that govern spatial representations and behavioural flexibility underlying goal-directed actions. Impairments of these astrocytic processes may therefore contribute to the spatial navigation, orientation and memory deficits characteristic of neurodegenerative disorders.

### Astrocyte pathophysiology in disorders of navigation and goal-oriented cognition

Dysregulation of astrocyte signalling is increasingly recognised as a key mechanism underlying the cognitive deficits observed in neurodegenerative disorders and AD (Brandebura et al., 2023). AD is characterised by a progressive decline in memory and higher cognitive functions, including spatial navigation, orientation, and abstract thinking (Coughlan et al., 2018). Astrocyte reactivity, defined as the remodelling of astrocytes in response to brain damage (Escartin et al., 2021), is present in AD; however, the role of this reactive state remains debated as reactive astrocytes have been associated with both protective and pathogenic effects depending on disease stage (Chun and Lee, 2018). Nonetheless, converging data indicate that molecular and cellular alterations in astrocytes actively participate in AD onset and progression.

Studies in both AD mouse models and postmortem human brains reveal that astrocytes surrounding amyloid-β (Aβ) deposits display altered expression and function of multiple receptors. For instance, astrocytic nicotinic-acetylcholine α7 receptor (α7-R), metabotropic glutamate receptor 5 (mGluR5), and P2Y_1_ purinergic receptors (P2Y_1_R), show enhanced Gq–IP₃–dependent signalling, exacerbating Ca^2+^ signalling dysregulation (Teaktong et al., 2003; Lim et al., 2013; Pirttimaki et al., 2013; Talantova et al., 2013; Delekate et al., 2014; Kim et al., 2025). These changes in the activity of different receptor pathways may alter astrocyte signalling and gliotransmission, causing downstream consequences for synaptic plasticity, memory and goal-directed behaviours.

Compromised astrocyte-neuron communication could imbalance excitation and inhibition, a hallmark of early AD, where altered oscillatory dynamics disrupt cognitive processing (Fontana et al., 2017; Pizzo et al., 2020). Both patients and mouse models of AD exhibit altered network oscillations and neuronal hyperexcitability, especially in the vicinity of Aβ deposits (Busche and Konnerth, 2015; Palop and Mucke, 2016). Astrocytes close to Aβ plaques have been observed to be hyperactive and with altered morphology (Guillemaud et al., 2020), displaying elevated resting Ca^2+^ levels, increased frequency and amplitude of Ca^2+^ transients, and long-range coordinated Ca^2+^ waves that are absent in healthy brains (Richards et al., 2003; Kuchibhotla et al., 2009; Delekate et al., 2014; Griffith et al., 2016; Fontana et al., 2017; Leparulo et al., 2019; Pizzo et al., 2020). Disrupted Ca^2+^ homeostasis, oxidative stress, and impaired glutamate uptake in turn compromise astrocyte-neuron communication, perturbing network synchrony (Jo et al., 2014; Verkhratsky et al., 2016; Santello et al., 2019; Nanclares et al., 2021), particularly within the hippocampus and cortical circuits supporting memory, spatial coding, and goal-directed navigation (Coughlan et al., 2018; Jun et al., 2020; Karunakaran, 2020; Nakano-Kobayashi et al., 2023).

Altered astrocyte recruitment and signalling are also likely to impair the plasticity mechanism underlying learning and memory consolidation. For example, astrocytic adenosine A2A receptors (A2ARs), which signal via Gs–cAMP pathways, are upregulated in AD (Orr et al., 2015). While A2ARs activation physiologically contributes to sleep-dependent synaptic downscaling and network homeostasis (Halassa et al., 2009), its aberrant upregulation and overactivation in AD (Orr et al., 2015; Lopes et al., 2021) may alter glutamate uptake and synaptic plasticity (Matos et al., 2013; Orr et al., 2015), thereby contributing to impaired memory retention, synaptic dysfunction, and spatial disorientation. In parallel, astrocytes exhibit enhanced synthesis and release of GABA, leading to a tonic inhibition of dentate gyrus granule cells and altered synaptic plasticity (Jo et al., 2014).

Astrocytic metabolic dysfunction further exacerbates cognitive decline. Compromised astrocyte-neuron metabolic coupling affects neuronal energy supply and synaptic functions (Harris et al., 2012; Demetrius et al., 2015; Pomilio et al., 2024). Notably, reduced astrocytic synthesis of L-serine leads to diminished D-serine availability and impaired NMDARs activation, resulting in deficits in synaptic plasticity, spatial memory, and learning (Le Douce et al., 2020).

It is therefore crucial to test interventions that restore astrocytic signalling or metabolic support to improve network function and cognition in AD. Several attempts have been made in different AD mouse models. Pharmacologically blocking astrocytic GABA release or GABA transporters restores long-term potentiation and rescues memory deficits (Jo et al., 2014; Li et al., 2019). Moreover, optogenetic manipulation of astrocytes, or viral-mediated expression of the stromal interaction molecule 1 (STIM1), a regulator of store-operated calcium entry, rescue astrocyte Ca^2+^ signalling, normalises neuronal activity and improves memory performance (Lee et al., 2023; Lia et al., 2023). It would be paramount to test the effectiveness and generality of these findings across the many AD mouse models showing astrocyte impairments, memory loss and spatial deficit (see table 1, Oddo et al., 2003; Oakley et al., 2006; Saito et al., 2014; Webster et al., 2014; Griffith et al., 2016; Boscia et al., 2017; Orr et al., 2018; Belfiore et al., 2019; Neuner et al., 2019; Ham et al., 2020; Heuer et al., 2020; Hamilton et al., 2023; Duggan et al., 2024; Gurdon et al., 2024; Qing et al., 2024).

In summary, these findings support the idea that astrocytes are central regulators of network stability, memory, navigation, and orientation in AD, identifying in the astrocyte-specific signalling pathways a promising new therapeutic target.

### Emerging concepts and future directions

Collectively, the evidence reported in this review supports a paradigm shift, from a neuro-centric view toward a framework where cognition emerges from dynamic neuron-astrocyte interactions. This shift is supported by growing evidence that astrocytes actively participate in synaptic plasticity, engram recruitment, spatial representations, and goal-directed behaviour (Mederos et al., 2021; Nagai, Yu et al., 2021b; Curreli et al., 2022; Doron et al., 2022; Sánchez Romero and Navarrete, 2026). A hypothesis that still needs to be experimentally tested is that astrocytes are integral components of the cellular networks that encode, store, and flexibly update cognitive information.

Despite rapid progress in this direction, several fundamental questions remain unresolved. It is still unclear how astrocytic and neuronal ensembles reciprocally interact to shape learning, memory, and navigation (Curreli et al., 2022; Doron et al., 2022; Williamson et al., 2024; Dewa et al., 2025; Serra et al., 2025), and whether astrocyte influence is primarily local or extends across distributed networks (Lines et al., 2024; Rupprecht et al., 2024). Likewise, it remains debated whether astrocytes can influence fast time-scale responses, such as those evoked by sensory stimuli (Stobart et al., 2018; Lines et al., 2024), or instead preferentially regulate slower processes through neuromodulation, synaptic homeostasis, and state-dependent network tuning (Rupprecht et al., 2024; Dewa et al., 2025). Additional open questions concern astrocyte heterogeneity: do distinct astrocyte subpopulations perform different functions within the same circuit (Chai et al., 2017; de Ceglia et al., 2023), and how do astrocyte roles vary across brain regions, behavioural contexts and pathology (Serrano-Pozo et al., 2024; Abiero et al., 2026)? Addressing these issues is essential to understand how astrocyte signalling, largely studied at cellular and microcircuit levels, affects cognition at systems and behavioural scales.

Historically, progress in this field has been constrained by the limited tools available to selectively monitor and manipulate astrocyte activity and astrocyte-neuron communication *in vivo*. However, advances in chemogenetic and optogenetic approaches, as well as the release of new signalling modulators and genetic methods, are rapidly expanding the experimental toolkit (Srinivasan et al., 2016; Losi et al., 2017; Mederos et al., 2019; Nagai, Bellafard et al., 2021a; Ollivier et al., 2024; Soto et al., 2024; Serra et al., 2025). These innovations will enable increasingly precise dissection of astrocyte function within defined circuits and behavioural contexts (Meron Asher and Goshen, 2025), further clarifying how astrocyte encodes information, and opening new avenues to establish causal links between astrocyte dynamics, circuit computations, and behavioural control.

Ultimately, decoding how astrocytes and neurons interact across brain-wide networks will not only provide insight into mechanisms of cognition but also reveal how they become disrupted in neurodegenerative disorders. In AD, key open questions include whether restoring astrocytic neuromodulation or synaptic pruning mechanisms (Dejanovic et al., 2022) could rescue circuit dysfunction and cognitive decline (Lee et al., 2023; Lia et al., 2023). Similarly, it would be crucial to understand which astrocytic signalling pathways (Poulot-Becq-Giraudon et al., 2026), and which astrocyte types (Serrano-Pozo et al., 2024), are most critical for maintaining network stability and memory consolidation in the ageing or diseased brain. Addressing these gaps will clarify astrocyte roles in pathogenesis and pave the way for astrocyte-targeted interventions to slow or prevent cognitive decline in AD and other forms of neurodegenerative disorders.
